# Subcutaneous device-free islet transplantation

**DOI:** 10.3389/fimmu.2023.1287182

**Published:** 2023-10-18

**Authors:** Xudong Zhou, Zhiran Xu, Yanqiu You, Wangrong Yang, BingZheng Feng, Yuwei Yang, Fujun Li, Jibing Chen, Hongjun Gao

**Affiliations:** ^1^ Guangxi University, Nanning, China; ^2^ Ruikang Hospital Affiliated to Guangxi University of Chinese Medicine, Nanning, China

**Keywords:** diabetes mellitus, subcutaneous islet transplantation, hydrogel, islet viability matrix, extracellular matrix diabetes mellitus, extracellular matrix

## Abstract

Diabetes mellitus is a chronic metabolic disease, characterized by high blood sugar levels; it affects more than 500 million individuals worldwide. Type 1 diabetes mellitus (T1DM) is results from insufficient insulin secretion by islets; its treatment requires lifelong use of insulin injections, which leads to a large economic burden on patients. Islet transplantation may be a promising effective treatment for T1DM. Clinically, this process currently involves directly infusing islet cells into the hepatic portal vein; however, transplantation at this site often elicits immediate blood-mediated inflammatory and acute immune responses. Subcutaneous islet transplantation is an attractive alternative to islet transplantation because it is simpler, demonstrates lower surgical complication risks, and enables graft monitoring and removal. In this article, we review the current methods of subcutaneous device-free islet transplantation. Recent subcutaneous islet transplantation techniques with high success rate have involved the use of bioengineering technology and biomaterial cotransplantation—including cell and cell growth factor co-transplantation and hydrogel– or simulated extracellular matrix–wrapped subcutaneous co-transplantation. In general, current subcutaneous device-free islet transplantation modalities can simplify the surgical process and improve the posttransplantation graft survival rate, thus aiding effective T1DM management.

## 1 Introduction

Diabetes mellitus, a complex, multi-etiology, chronic metabolic disease, has affected more than 500 million individuals worldwide (1). By 2045, this number is expected to reach 693 million (2). Type 1 diabetes mellitus (T1DM) (3) is an endocrine disorder, whereby pancreatic β cells undergo autoimmune destruction and eventually stop producing insulin; this eventually leads to extreme insulin shortage, followed by several metabolic dysfunctions, specifically poor glucose homeostasis. Diabetes mellitus was once a fatal disease; nevertheless, the discovery of insulin in 1921 made it a treatable, chronic condition. Leonard Thompson, a 14-year-old patient with severe diabetes at the Toronto General Hospital, was the first human to be treated with insulin injections; however, the clinical trial failed (4). Although strict glucose management with insulin can prevent or even eliminate the long-term consequences of diabetes mellitus, an insulin overdose may lead to fatal hypoglycemia. Moreover, several complications (5) are associated with diabetes; they include blindness, stroke, renal failure, heart attack, and lower limb amputation due to severe gangrene.

Strict glucose management is linked to more hypoglycemic episodes, particularly in individuals with severe disease. Many individuals with T1DM lose awareness of hypoglycemia to some extent and therefore cannot sense it. Hypoglycemia unawareness, linked to up to 10% of T1DM mortality, can result in unconsciousness or the inability to wake up from sleep (i.e., dead-in-bed syndrome) (6). Patients with T1DM require continuous glucose monitoring and insulin infusion devices for lifelong insulin delivery; this treatment places a serious clinical and financial burden on patients. Therefore, newer, inexpensive treatment modalities are urgently needed.

Pancreatic islets can regulate blood glucose more precisely than current artificial pancreas systems; therefore, islet transplantation has great potential for T1DM management (7). However, the shortage of pancreatic donors poses a significant obstacle for type 1 diabetes patients, as well as hindering the clinical application of islet transplantation. Recently cell-replacement therapies using functional insulin-producing pancreatic β cells generated from stem cell differentiation or transdifferentiation offer potential treatment options (8). However, these approaches still have several limitations compared to somatic islets derived from the same individual. Islets contain multiple cell types that work together to maintain glucose metabolism homeostasis, which cannot be fully replicated by a single β cell type (9). Moreover, β cells generated from stem cells may have different gene expression patterns or mutations compared to somatic β cells, and some may still retain pluripotency potential, increasing the risk of tumor development. Additionally, these cells may elicit unwanted immune responses. Therefore, we can consider other approaches to tackle the shortage of islet donors. This study (10) aimed to optimize the expansion of pancreatic islets *in vitro* by examining the chemicals and factors used in the culture of various organoids. These included organoids derived from pancreatic duct cells, cholangiocytes, hepatocytes, stem cells, and fibroblasts. Based on their functions, these chemicals and factors were classified into three groups: islet identity, cell division and proliferation, and cell renewal and regeneration. Through screening different combinations of compounds and factors, the researchers developed a medium known as pancreatic islet expansion medium (PIEM). PIEM, consisting of 12 chemicals, hormones, and essential nutrients like nicotinamide, B27, and GlutaMAX, demonstrated robust capabilities in supporting the *in vitro* expansion of dispersed islet clusters obtained from pregnant mice or wild-type rats. The development of PIEM is an exciting breakthrough in the field of regenerative medicine as it paves the way for the efficient expansion of pancreatic islets *in vitro*. This not only saves time and resources, but it also offers hope for overcoming the limited availability of donor pancreatic tissue, a crucial barrier in islet transplantation therapy. The findings of this study (10) signify a major step towards the effective treatment of diabetes, and could potentially revolutionize the field of cell-replacement therapies. Islet transplantation involves islet cell extraction from a donor by using biotechnological methods, followed by their transfusion into a patient’s body through clinical techniques; currently, the most widely used transfusion technique is portal vein islet transplantation. The islet microenvironment refers to the complex external conditions under which the islet cells survive. The graft survival rate depends mainly on whether the islet microenvironment provides sufficient oxygen and nutrients to the islets. Therefore, islet microenvironment reconstruction and transplant site selection are crucial considerations for islet transplantation in patients with T1DM.

### 1.1 Islet microenvironment and the extracellular matrix

The islet cell mass contains various cells, such as insulin-producing β cells, glucagon-producing α cells, somatostatin-producing δ cells, and pancreatic polypeptide-producing γ cells (11). Moreover, the microenvironment of islet cells includes many innervations, endocrine cells, and macrophages. The endocrine cells behave differently depending on the species in terms of how they secrete hormones. Creating an *in vitro* pancreatic islet organoid, incorporating all the essential cell types responsible for regulating islet functions, is vital for adequately substituting the role of natural islets. Rats and humans demonstrated differences in islet cell distribution; in rats, the islet cells are wrapped by other cells and mainly concentrated in the center of the islets, whereas they are evenly distributed in humans (12). The primary function of these cells is to maintain glucose homeostasis, accomplished through paracrine and autocrine signaling between the cells. Adhesion molecules have a key function in coordinating cell activities in the islet microenvironment. In fact, any blockage or absence of an adhesion molecule hinders glucose homeostasis and obstructs cooperative actions (13). Another crucial component of the islet microenvironment is the vasculature. In the pancreas, the islet mass is very low, accounting for <1% of the total pancreas mass; nevertheless, all islets receive >10% of the blood flow of the entire pancreatic organ; in other words, the islets have an extremely rich microvascular network, which provides sufficient oxygen and nutrients (14, 15). The pancreas has two types of capillaries: one supplies blood to the exocrine cells, and the other supplies it to the endocrine cells. Endocrine capillaries are significantly denser, stronger, and thicker than exocrine capillaries. Numerous capillaries provide a large amount of oxygen and nutrients to the islets and aid them in sensing blood sugar levels relatively rapidly and accurately and then maintaining the body’s blood sugar levels dynamic and stable by secreting insulin and glucagon (16). The presence of endocrine cells expressing high levels of vascular endothelial growth factor (VEGF) A, which promotes neuronal dispersion, development, and function in the islet microenvironment.

The extracellular matrix (ECM) is extremely important for islet cell growth and reproduction and is an essential part of the islet microenvironment. Within the islet architecture, ECM fibers divide exocrine and endocrine cells. The islet ECM components include collagen, fibronectin, laminin, glycosaminoglycans, and fibrin, which are critical for β-cell growth. A thorough morphological investigation revealed that a capsule containing fibroblasts and collagen fibers, which surrounds islet cells, is incompletely formed. A proteolytic enzyme disrupts the ECM during pancreatic islet isolation, which increases stress in the islet cells significantly. Islet stress can be minimized through the application of a system mimicking the ECM scaffold to the islets after isolation and transplantation so as to retain and sustain islet viability and functionality (17, 18). This may ultimately improve graft survival after islet transplantation.

### 1.2 Clinically alternative extrahepatic transplantation sites

#### 1.2.1 Omentum

The omentum is considered a prime location for islet transplantation due to its excellent neovascularization abilities, tissue regeneration properties, hematostatic characteristics, and immune-privileged status. The omentum, located in the abdomen, is mainly composed of a large adipose tissue layer (19). It can store abundant adipose-derived stem cells and provide a protective environment for immune cells such as macrophages as well as B, T, and mast cells, which can help protect vital organ activities. The hOMING (i.e., h-Omental Matrix Islet filliNG) transplantation technique involves injecting the graft within the omental tissue layers, thus enhancing islet implantation and survival (20). Furthermore, there is study have explored the use of biocompatible plasma-thrombin gel for omental islet transplantation as a potential clinical approach (20). This study emphasize the technique’s benefits, including improved islet cell survival and protection against immune rejection, which ultimately translates to a reduced dependence on insulin therapy and an improved quality of life for recipients. Although challenges exist, there is optimism surrounding the potential for improvements in omental islet transplantation survival rates through the use of biomedical engineering techniques and skill sets. Furthermore, the wide-scale application of biopolymer materials in transplantation technology, such as hydrogels and polylactic acid, may also contribute to further successes in this area. By conducting additional clinical and theoretical research, the progress and efficacy of omental islet transplantation technology can be further advanced. This will result in greater treatment options for diabetic patients and an increased quality of life.

The primary goal of studying islet transplantation in the omentum is to reinstate insulin production in patients with diabetes to eventually eradicate the need for immunosuppressive medications (19). The omentum provides an advantageous microenvironment and immune privilege, which make it a promising option for islet transplantation. Despite intramuscular transplantation of islets being a convenient option that shows promise in terms of vascularization, it demonstrates significant challenges due to a strong immune response and limited life expectancy of allogeneic islets. However, using biocompatible materials with immune-modulating strategies may aid in overcoming these limitations.

#### 1.2.2 Intramuscular

Muscles have been extensively used as a transplantation site, particularly for the autotransplantation of the parathyroid glands; the technique has demonstrated effective long-term outcomes with minimal side effects. This success has sparked interest in considering muscles as a potential site for islet transplantation.

Intramuscular transplantation offers several advantages. First, muscles can develop a dense vasculature, much similar to that during exercise, resulting in oxygen tension levels that closely resemble those in the native pancreas (21). Second, the surgical procedure for intramuscular implantation is relatively simple and can be performed under local anesthesia with few risks and complications. Third, islets can be transplanted in multiple muscle sites, enabling multiple and repeated implantation and explantation of the transplanted tissue. This enhances the flexibility of the transplantation approach. This article (22) presents the intramuscular autotransplantation of islets in a 7-year-old child who had severe hereditary pancreatitis and underwent total pancreatectomy (**Table 1**). The procedure aimed to alleviate the child’s diabetes symptoms, and the study included a two-year follow-up. The results revealed a significant improvement in the patient’s blood sugar levels after transplantation, with only a minimal dosage of islets utilized. However, the restricted space within the muscle limits the number of islets that can be implanted, leading to some patients still requiring insulin therapy. Furthermore, there has been clinical study on intramuscular islet allograft transplantation for type 1 diabetes mellitus treatment. These studies involved transplanting islets from a donor into the patient’s muscle tissue and monitoring their effectiveness. The findings demonstrated that the transplanted islets functioned effectively in the muscle tissue, resulting in improved glycemic control in the patients. This suggests that intramuscular islet allograft transplantation shows promise as a viable treatment option for type 1 diabetes mellitus.

**Table 1:** Clinically alternative extrahepatic transplantation sites.

Clinically alternative extrahepatic transplantation sites	Graft source	Receptor	Transplant equivalents for per recipient	Description	Result	References
Omentum	Lewis rats	Lewis rats	Marginal,4 pancreatic islets/g; standard,10 pancreatic islets/g.	Animals underwent transplantation of 4 or 10pancreatic islets per gram of body weight (861 ± 50 pancreatic islets per animal or 2085 ± 76 pancreatic islets per animal) into the greater omentum using the plasma-thrombin bioscaffold.	Transplantation of 4 or 10 islets maintained normoglycemia for 100 days, and hyperglycemia recurred after removal of the graft.	(108)
	Cynomolgus monkeys	Cynomolgus monkeys	~16730IEQ/Kg	Islets were resuspended into 3 mL autologous recipient’s plasma supplemented with heparin (70 units/kg of recipient body weight) when ready for transplantation. Then islets was dripped onto the omentum surface and immobilized on the omentum by topical recombinant thrombin layered over the islet slurry; followed by another layer of autologous plasma to create a degradable biologic fibrin matrix; the omentum was then folded onto itself and held in place by the thrombin-induced fibrin glue.	After transplantation, normal blood glucose was maintained for 90 days in uninfected animal models and restored stable insulin and C-peptide levels that were comparable with the pre-STZ levels.	(109)
	C57BL/6J female mice (10 to 14 weeks old)	C57BL/6J male mice (10 to 14 weeks old);	~600IEQ	Study comparing three leading extrahepatic islet transplantation site(subcutaneous, smal bowel mesentery.epididymal fat pad) for syntheticvasculogenic hydrogel-based islettransplantation	The islets with the vasculogenic hydrogel transplantedinto the epididymal fat pad achieved normoglycemiawithin two weeks, for more than 35 days up to 100 days(approximately 75% of recipients)	(110)
	Human islet	One man and one woman with a long history of type 1 diabetes	~12,350 and 5,350 islet equivalents per kilogram	The recipients received 12,350 and 5,350 islet equivalents per kilogram that were mixed with autologous plasma, seeded during a laparoscopic procedure on the omentum, overlaid with human thrombin solution, and fixed by flapping the omentum over.	During a 9-month follow-up, neither patient experienced any moderate or severe hypoglycemia. Their glucose control significantly improved, insulin dose decreased by approximately 50%, and C-peptide at 1 year was 0.22 and 0.14 pmol/mL, respectively.	(111)
Intramuscular	Lewis rats	Lewis rats	~3000 pancreatic islets	In six rats, 3,000 islets were transplanted into gastrocnemius muscle flaps, and in the other six rats, the same number of islets were transplanted into the gastrocnemius muscle.	In the muscle flap group, blood glucose levels significantly decreased after islet transplantation(27 days). Blood glucose levels were significantly different between the two groups at 3 weeks after transplantation. The muscle flap group showed nearly normoglycemic results upon the glucose tolerance test, whereas the muscle group was hyperglycemic.	(112)
	C57BL/6 mice	Adult male C57BL/6 mice	~200 islets	Muscle and the greater omentum are two alternative implantation sites. Then they evaluated the functional outcome after islet transplantation to muscle and omentum.	Syngeneic transplantation of 200 islets reversed diabetes in 9 out of 11 (82%) animals after intramuscular implantation and in 9 out of 10 (90%) after implantation into the omentum.	(113)
	Human islet	7 years old patient	~160 000 islet equivalents (6400 islet/kg)	The islets were transplanted under the effect of general anesthesia. The total islet volume of 1.25 mL was then transplanted in eight aliquots between the fibers of the brachioradialis muscle of the right forearm.	Observational period lasted for two years, and the patientachieved better quality of life but insulin-independencewas not achieved.	(22)

Although muscles represent a convenient islet transplantation site, their use poses some limitations. The intramuscular environment is hypoxic, resembling that of subcutaneous tissues, which lack neovascularization. As such, relevant studies have not been able to achieve or maintain normoglycemia over the long term. Furthermore, transplanting islets into muscles leads to reduced efficacy when compared with transplanting them into other sites such as the liver or kidneys.

#### 1.2.3 Subcutaneous space

Subcutaneous transplantation of islets (23) is simple, poses low complication risk, and demonstrates ease of access for monitoring and retrieval. However, a lack of vasculature in the subcutaneous space hinders nutrient and oxygen supply, affecting the effectiveness of transplantation. To address this limitation, bioengineering techniques and biomaterials as well as strategies to induce angiogenesis are essential to support transplanted islet engraftment and achieve normal blood glucose levels.

Various islet transplantation technologies that use biocompatible biomaterials have emerged. These biomaterials encompass hydrogels constructed using natural polymers such as collagen, fibronectin, fibrin, laminin, and alginate, as well as synthetic polymers such as polyethylene glycol (PEG), polyglycolic acid, polyvinyl alcohol, and dextran (24–27). Manipulation of these biomaterials to customize their mechanical, biological, and biochemical properties enables precise regulation of characteristics such as stiffness, growth factor and bioactive signal integration, degradability, enzyme sensitivity, and cell adhesion. Moreover, these biomaterials possess proangiogenic attributes, thereby facilitating blood vessel development in the subcutaneous milieu.

These engineered biomaterials can not only accommodate pancreatic islets but also host other types of cells, such as human adipose-derived stem cells (hADSCs), which have exhibited promise in augmenting the outcomes of transplantation. These biomaterials can be used in the microencapsulation and macroencapsulation processes, which involve coating pancreatic islets with the biomaterials. Alternatively, they can be designed as implantable scaffolds, which support the encapsulated islets and assist in nutrient exchange. Notably, hADSCs can form a physical barrier, safeguarding the islets against immune cells.

Challenges related transplantation of encapsulated islets at subcutaneous sites, however, persist. This is because a higher number of islets (often two to five times more) may be required compared with naked transplantation so as to achieve normal blood glucose levels (1). Combining subcutaneous transplantation with angiogenic and immune-modulating biomaterials shows promise, but the long-term sustained outcomes have yet to be definitively demonstrated in clinical studies compared to intraportal transplantation.

A major reason for the suboptimal performance of subcutaneous transplantation is the immune response, which leads to fibrotic cell encapsulation around the islets, limiting their access to oxygen and nutrients. Immune-protective devices that physically separate transplanted islets from immune cells may not fully account for the impact of diffusible immune factors on islet function (28). Therefore, further research exploring advanced biomaterials with angiogenic and immune-modulating properties to achieve long-term survival of transplanted islets in the subcutaneous space is needed.

**Figure 1:**
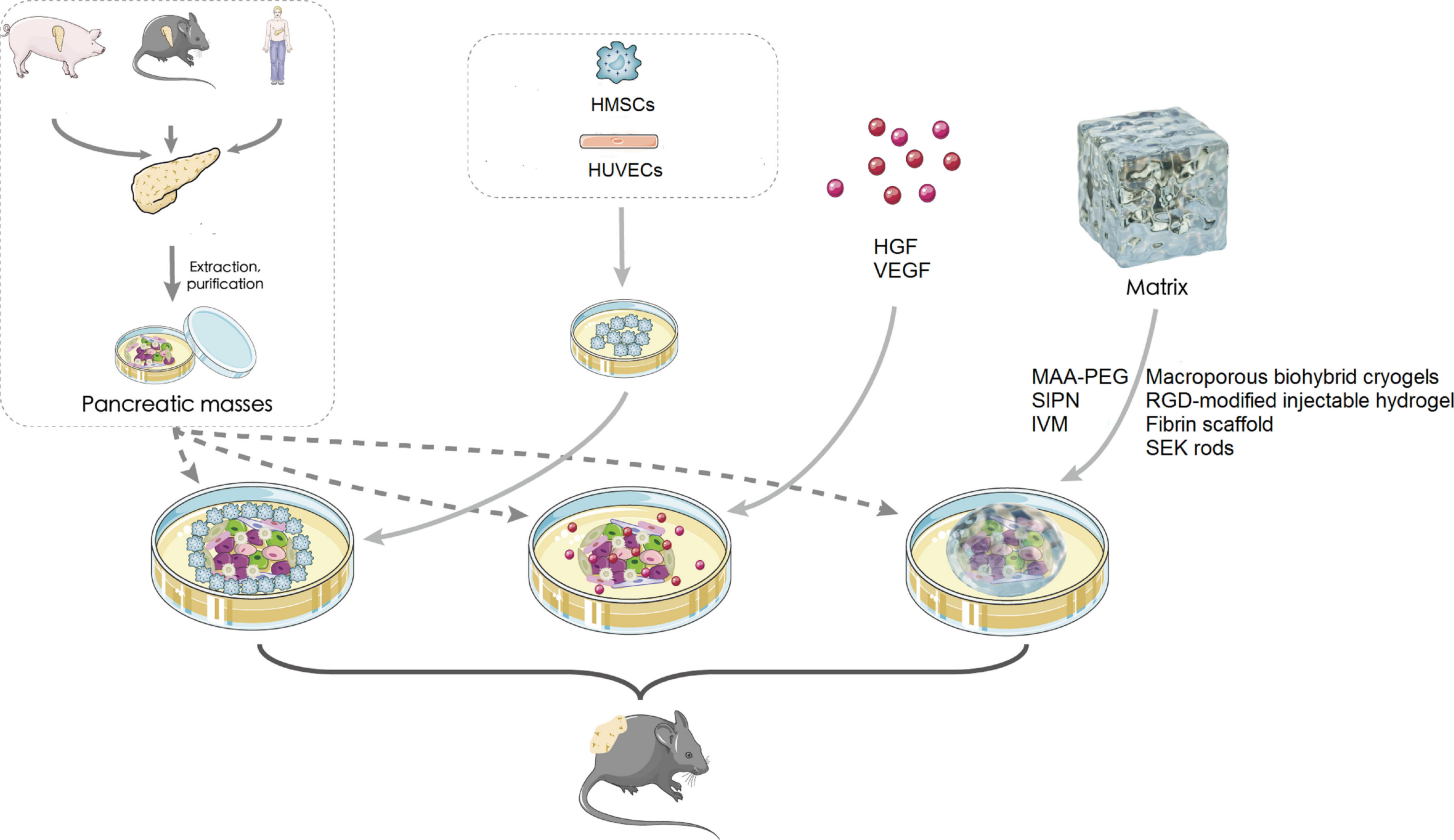
Subcutaneous islet transplantation. HMSC, human mesenchymal stem cell; HUVEC, human umbilical vein endothelial cell; HGF, hepatocyte growth factor; VEGF, vascular endothelial growth factor; MAA-PEG, methacrylic acid-polyethylene glycol; SEK, agarose-SEK-1005; IM, islet viability matrix; SIPN, semi-interpenetrating polymer network.

Herein, we mainly discuss three subcutaneous device-free islet transplantation methods (**Figure 1**), which promote the survival rate of islets after transplantation: islet cotransplantation with cells, cell growth factors, and hydrogels or ECM.

## 2 Coculture and cotransplantation of islets with other cells

### 2.1 Coculture and cotransplantation with mesenchymal stem cells

Mesenchymal stem cells (MSCs) are self-renewing cells, which form a crucial cellular component of the body. They are mainly found in the bone marrow and account for 0.001%–0.01% of its cell population (29). MSCs can also be isolated and extracted from other tissues (e.g., adipose tissue and umbilical cord blood), and their ability to self-renew is not affected by the extraction process.

MSCs also elicit a reparative effect by moving to sites of damage and generating paracrine substances that impact local cell movement, growth, and viability. MSCs can not only improve transplantation outcomes but also regulate the body’s immune system and promote angiogenesis through trophic factor secretion (30). The trophic factors secreted by MSCs include hepatocyte growth factor (HGF) (31), transforming growth factor (TGF) β (32), VEGF, interleukin (IL) 6 (33), IL-10 (34), annexinA1 (35), indoleamine-2,3-dioxygenase, and prostaglandin E2 (36). These factors play important roles in the repair of damage and promotion of angiogenesis, inflammation resolution, and immunomodulation.

Several studies have reported that MSCs promote tissue repair and regeneration in conditions such as diabetic wounds, cardiac infarction, and liver fibrosis (37–39). MSCs have also been used to modulate the immune system in cell therapy for autoimmune diseases and inflammatory disorders. By inhibiting effector T-cell proliferation, promoting regulatory T-cell generation and function, and suppressing B-cell activation, MSCs can ameliorate autoimmune diseases and reduce inflammation.

Because they can secrete proangiogenic factors such as VEGF, platelet-derived growth factor (PDGF), and TGF-β, MSCs can also promote angiogenesis (i.e., promote new blood vessel formation) and endothelial progenitor cell recruitment. These angiogenic effects, which result in nutrient and oxygen supply, waste removal, and immune cell trafficking, play a critical role in tissue regeneration.

In general, MSCs can be used as a promising therapeutic component because of their potential for promoting tissue regeneration and modulating the immune system through trophic factor secretion. Further research elucidating the mechanisms of action of these factors and optimizing their use in clinical applications is required.

#### 2.1.1 Coculture and cotransplantation with adipotic MSCs

In a study, the addition of adipotic MSCs (adMSCs) did not appear to improve the capacity of transplanted islets to restore normoglycemia in mice with diabetes. In subcutaneous transplantation of the islet module (adMSC in), only one-third of the SCID/bg mice receptors returned to normal levels (40). In the early stages of islet transplantation, adMSCs may not provide any vascularization advantage in the presence of the islets given that islets also release proangiogenic growth factors (e.g., VEGF-A) in response to hypoxia. Moreover, the added adMSCs compete with the islets for oxygen and nutrients, resulting in unsatisfactory outcomes (40). However, if the number of adMSCs is controlled appropriately, they can provide more health benefits. Gamble et al. investigated the effects of coculturing murine islets with human adMSCs both *in vitro* and *in vivo*. After coculturing the islets with adMSCs for 48 h, the authors observed decreased cell death, improved viability indicated by intact membrane integrity, enhanced insulin secretion in response to glucose stimulation, and increased euglycemic rates in mouse islet grafts with adMSCs compared with islets cultured and transplanted alone. The authors also examined the results of coculturing human adMSCs with murine and human islets for 48 h by using islet-to-adMSC ratios of 1:300 and 1:2000. The findings indicated that the islet-to-adMSC ratio of 1:2000 led to better outcomes than that of 1:300 (41). To lessen this competition and preserve the potential regeneration abilities of these cells, a study suggested reducing adMSC density in pseudo-islets by a factor of 10 (42). *In vitro* glucose stimulation experiments demonstrated that insulin secretion from the pseudo-islets with adMSCs was stronger than that from natural islets. However, at day 31, the pseudo-islets with adMSCs did not significantly improve the vasculature at the transplantation site or the number of β-cells *in vivo* relative to the native islets. The pseudo-islets exhibited a greater ability to secrete insulin, leading to faster recovery of blood glucose, than did the natural islets (42). Notably, the addition of adMSCs to native islet modules at levels 10-fold lower than in the aforementioned studies led to a negative impact on islet function.

Islet modules containing adMSCs are well vascularized; this may be due to islet disaggregation modifying the environment of pseudo-islets, resulting in adMSC secretion of paracrine factors distinct from those incorporated in the original islet modules (43). Furthermore, the ability to control the size of the pseudo-islets is crucial because the pancreatic islet size affects hypoxic core development and transplantation efficacy. Notably, pseudo-islets embedded in adMSCs produce significantly smaller constructs than HUVECs seeded on the surface (42). Furthermore, Kuppan et al. (44) demonstrated that permanent neonatal porcine islet (NPI) engraftment occurs after cotransplantation of NPI (3000 islet equivalent quantity [IEQ]) and adMSCs (2 × 10^6^) into the subcutaneous device-free region or kidney capsule. Compared with NPIs alone, the cotransplantation of adMSCs and NPIs significantly improved subcutaneous graft site angiogenesis, thereby accelerating the metabolic rate at the site. Immunodeficient B6.129S7-Rag1tm1Mom/J mice recipients who received cotransplanted adMSCs and NPIs demonstrated a shorter time to blood glucose normalization, along with lower fasting and nonfasting blood glucose levels, than those transplanted with NPIs alone (44). Finally, they obtained ideal experimental results, with a shorter time to reach normal blood glucose levels, as well as increased glucose clearance and total insulin content. Transplanting MSCs has various benefits in regenerative and reparative medicine, but it also poses risks (45). These risks, such as tumor formation, undesired immune reactions, and transmission of adventitious agents, can occur regardless of therapeutic intent. Such risks depend on different factors, including the type of stem cells used, their differentiation status, proliferation capability, administration route, intended location, and culture conditions and genetic manipulation strategies. To minimize these risks, it’s crucial to thoroughly characterize and manufacture the cellular product according to regulatory standards that exceed the minimum requirements. In a study, cotransplantation of MSCs extracted from adipose tissue or bone marrow with islet cells could lower blood sugar levels and improve transplantation success rates (29). Moreover, adMSC–islet cell cotransplant recipients demonstrated an increase in the levels of both total cellular graft insulin and stimulated porcine insulin. Research on the production of sheets in which MSCs and fibroblasts play supportive roles has demonstrated effective results (46). Adipose-derived stem cells can restore euglycemia with a small number of islets, suggesting that using the cotransplant is a viable, practical approach for the cell-based treatment of T1DM (47). The specific mechanisms through which MSCs improve glycemic control remain unclear. However, some studies have suggested that the secretion of some trophic factors may play a role in this process. Although MSCs can be derived from different tissues in the body, most studies have indicated that the secretion of trophic factors, which promote islet revascularization, is responsible for improving metabolic graft function (8).

#### 2.1.2 Coculture and cotransplantation with bone marrow–derived MSCs

Cotransplantation of rodent bone marrow–derived MSCs (BMSCs) with islets was noted to reduce blood glucose levels (48). Although the islet transplant site in the aforementioned study was the renal capsule, the mechanisms of action of the cells and the trophic factors remained unchanged. However, the role of BMSCs in a subcutaneous transplant site is debatable. A study reported that the recipients of subcutaneous cotransplantation of rodent islets and MSCs demonstrated increased glucose tolerance (49). The length of time during which individuals with T1DM are in a hyperglycemic state is proportional to the risk of diabetic complications; therefore, with increased glucose tolerance, the time under hyperglycemia decreases—which is of great clinical significance. Wang et al. (30) discovered that low-grade chimerism lacked the beneficial hypoglycemic effects of a complete chimerism; they also investigated the anti-inflammatory impact of MSCs on islet engraftment and noted that both the renal subcapsular and subcutaneous allogeneic islets were protected by full donor chimerism. To reestablish normoglycemia, transplanting many additional islets through the subcutaneous location and quickly reaching full donor chimerism was necessary.

#### 2.1.3 Coculture and cotransplantation with human umbilical cord mesenchymal stem cells

Yu et al. (50) recently demonstrated that HUCMSCs, human venous endothelial cells, and MIN6 can be fabricated into three-dimensional (3D) cell spheroids, which could be cotransplanted with islets. HUCMSCs can modify their surroundings *via* paracrine signaling; thus, they are a potent tool for the creation of a proregenerative environment. When transplanted into ischemic tissues, endothelial cells undergo a transformation—from forming simple tubular structures to developing into functional vasculature. This process is facilitated by trophic and proangiogenic factor release through paracrine signaling by HUCMSCs or the differentiation of HUCMSCs into perivascular cells.

A study (50) revealed that 3D stem cell spheroids, comprising HUCMSCs, human umbilical vein endothelial cells, and MIN6 cells, produce diverse paracrine factors, which strongly enhance vascularization and increase the survival rate of cells transplanted into subcutaneous sites. To determine the optimal density of β cells for subcutaneous transplantation, three MIN6 cell doses were examined: compared with 2 × 10^6^ MIN6 cells, 1 × 10^6^ MIN6 cells resulted in a stronger bioluminescent signal 2 weeks after transplantation, indicating that 1 × 10^6^ MIN6 cells demonstrate higher viability at the transplant site. The higher cell mortality observed in the high-cell density group was attributable to heightened competition for limited oxygen and nutrient resources in subcutaneous sites with inadequate vascularization. The MSC secretory group is complex, which means that no single factor can fully achieve the benefits of MSC co-culture in islet transplantation. However, pre-incubating islets with a specific mixture of MSC secretion factors before transplantation is a simple and potentially effective approach (51). This method can protect β cells from hypoxia and inflammation immediately after transplantation, as well as reduce competition for oxygen and nutrients from co-transplanted cells. Overall, this approach holds promise for improving the outcomes of clinical human islet transplantation. This coincides with our previous description: patients who receive islet transplantation for T1DM treatment might demonstrate improved insulin independence after codelivery of islet cells with 3D stem cell spheroids. However, because of their great sensitivity to microenvironmental changes, isolating cells from harvested pancreatic islets for further modification is impractical. In contrast, the 3D MSC/HUVEC spheroids, created by Yu et al., can be directly cotransplanted with intact islets.

HUCMSCs elicit various advantages and are considered a promising MSC source; they are obtained from umbilical cords, which are typically discarded as medical waste, thus avoiding ethical concerns. HUCMSCs collection is a non-invasive, ex vivo procedure, which carries no risk of infection or discomfort for donors—which makes it a relatively acceptable option. Furthermore, HUCMSCs demonstrated a stronger proliferation capacity than BMSCs *in vitro*; as such, they demonstrate an increase in cell yield and adequate clinical supply. Moreover, HUCMSCs expanded *in vitro* form a relatively homogeneous population, which may facilitate standardization in the production of MSC products (52).

### 2.2 Coculture and cotransplantation with HUVECs

Vascular endothelial cells have many physiological roles; they are crucial for maintaining vascular tone, controlling blood pressure, preventing thrombosis, and neovascularization. Their monolayer flat cells orient longitudinally on the surface of the vascular intimum (53).

HUVECs represent one of the most popular endothelial cell types. Modular tissue engineering, incorporating HUVECs, can produce a subcutaneous, vascularized bed, which permits pancreatic islet transplantation (40). Injection of 750 rat islet equivalents embedded in endothelialized collagen modules effectively restored and maintained normoglycemia in SCID/beige mice with streptozotocin-induced diabetes for a period of 21 days. In contrast, 750 free islets did not affect blood glucose levels. This difference is attributable to the process of islet revascularization and integration facilitated by the endothelialized collagen modules. However, the inflammation that results in vascularization might impede islet function, which poses another major barrier to islet transplantation angiogenesis. Notably, despite the differences being nonsignificant, the islets embedded in modules (with or without HUVECs) demonstrated a greater fraction of M2-like macrophages (CD206 + MHC II) at day 7 compared with the no-islet control (40). The survival and proliferation of β cells are positively affected by M2-like macrophages (54). The stronger inclination toward M2 macrophages may contribute to islet survival and vessel integration. Furthermore, the presence of HUVECs in the construct provides protection against collagen degradation, allowing the construct to remain intact and retrievable. This retrievability feature is a significant clinical consideration for further research using alternative insulin-producing cells, such as stem cell–derived pancreatic progenitor cells. Takaichi et al. (55) recently established β-cell spheroids using human induced pluripotent stem cell (hiPS)-derived cells, normal human dermal fibroblasts, and HUVECs. *In vitro*, vascularized hiPS-cell spheroid tissue demonstrated a considerable increase in insulin secretion. When subcutaneously implanted into NOD/SCID mice with T1DM, this tissue considerably reduced blood glucose levels. The explanted vascularized hiPS-cell spheroid tissue also contained host mouse vasculature. These results suggested that a promising regenerative therapy in patients with T1DM patients might involve subcutaneous implantation of 3D vascularized spheroid tissue using stem cell–derived cells.

## 3 Cotransplantation of islets with cell growth factors

### 3.1 Cotransplantation with HGF

Subcutaneous islet transplantation is associated with several problems, including postinfusion thrombosis and rapid blood-mediated inflammatory response, leading to the death of the transplanted islets. HGF plays a role in the regulation of inflammation by reducing the production of the proinflammatory cytokine IL-6 and increasing the levels of the anti-inflammatory cytokine IL-10 in bone marrow–derived macrophages (56). A study (57) specifically demonstrated that HGF can effectively alleviate acute inflammation in lipopolysaccharide-stimulated bone marrow–derived macrophages. HGF can aid in breaking the inflammatory cycle between macrophages and adipocytes by decreasing proinflammatory cytokine production and increasing adiponectin secretion. Under insulin resistance, insulin signaling pathways undergo various alterations, resulting in decreased insulin activity. The binding of HGF and the c-Met receptor and the formation of the c-Met–INSR complex leads to the recruitment of insulin receptor substrates (IRS-1 and IRS-2), which enhance insulin signaling at the molecular level (58). Taken together, these mechanisms amplify HGF-induced signaling, thereby favoring the blood glucose stabilization processes. Therefore, HGF is linked to diabetes.

HGF also promotes vascularization at the subcutaneous site (58). In a recent study (59), ectopic expression of HGF-related genes increased the number and size of β cells and islets, as well as the total islet mass, in rats. HGF also improves islet revascularization and reduces cell death in apoptotic islets. The effects of HGF on islets have been demonstrated in several *in vivo* and *in vitro* studies (60). For instance, HGF can boost the number and quality of islet cells *in vivo* and enhance human islet activity and longevity after transplantation. By using a new formulation that coencapsulates HGF and islets, Yang et al. (61) demonstrated the viability of prevascularization-free primary subcutaneous transplantation. *In vitro*, HGF boosted islet survival in 1% oxygen culture and preserved glucose-stimulated insulin secretion. In contrast, the islets treated with 1% oxygen but not HGF tended to die and release a considerable amount of insulin.

HGF induces vascularization in the subcutaneous area and improves islet survival through protein kinase B/Akt, whereas the disruption of HGF/c-Met signaling can lead to β-cell death. Moreover, the HGF-induced PI-3K/Akt activation attenuates hypoxia-induced intracellular oxidative stress and apoptosis. This is consistent with our earlier description of how HGF affects insulin release. In an *in vivo* study, 600 IEQ and HGF were encapsulated in islet–chitosan gel–ethylene vinyl alcohol bag and then subcutaneously transplanted into C57BL/6 mice. During the 28 days of observation, these mice demonstrated a blood glucose level that remained consistently lower than mice in the islet-alone group but consistently higher than untreated healthy mice. The results demonstrated that coencapsulating islet + HGF devices significantly enhanced the outcomes of primary subcutaneous islet transplantation. In the future, the HGF coencapsulating islet device might be a viable choice for T1DM therapy in terms of therapeutic replacement and regeneration.

### 3.2 Cotransplantation of islets with VEGF

The subcutaneous space is a desirable alternative to other transplantation sites because of its accessibility, imaging potential, and retrievability (62). However, the restricted vascularization and low oxygen tension at the subcutaneous space are key issues (63). Consequently, islet transplants with auxiliary devices and drugs have been created (64). However, conventionally encapsulated islets do not have adequate access to vascular arteries, nutrition, or growth hormones (65).

To stimulate angiogenesis, VEGF-A is frequently employed. Pancreatic islets and microvascular endothelial cells have a close and essential connection, with endothelial cells playing a critical role in supporting pancreatic β-cell function. Angiogenic factors such as VEGF-A, which aid in the survival and growth of adjacent endothelial cells, are released by β cells. These endothelial cells, in turn, contribute to efficient glucose sensing and β-cell–secreted insulin transportation. However, prolonged overexpression of β-cell-specific VEGF-A can lead to β-cell apoptosis and impaired tolerance to glucose. In contrast, slight overexpression of β-cell-specific VEGF-A in adult mice promotes the replication of β cells. Therefore, it is crucial for VEGF-A to maintain a narrow physiological range and preserve the balance and functionality of pancreatic islets. Thus, adding VEGF-A during the early stages of transplantation can effectively enhance graft survival and large-scale replication after islet transplantation (66). Aamodt et al. (67) reported that compared with mature islets, developing islets have a much greater need for endothelial cell recruitment and subsequent stimulation of endocrine cell proliferation.

Yang et al. (61) created VEGF-modified polyvinyl alcohol/SiO_2_ composite nanofibers (SiO_2_-VEGFs) for subcutaneous islet transplantation after device-free surgery. The authors reported that SiO_2_-VEGFs were more angiogenic than silicone alone and caused a milder inflammatory reaction in response to foreign bodies than nylon. Compared with device-free surgery alone, SiO_2_-VEGF with device-free surgery improved the function of subcutaneously transplanted islets. The results also indicated that SiO_2_ nanofibers might stimulate neovascularization. SiO_2_-VEGFs might accelerate vascularization. *In vitro* studies have shown that collagen tissues, when used along with oligomers to capture islets, can improve islet survival and insulin secretion. Therefore, the collagen content itself, rather than fibrotic encapsulation alone, can aid in reestablishing the islet matrix (68). Moreover, collagen I expression increased around the transplantation site in the SiO_2_-VEGF group, indicating that cotransplantation of islets with VEGF potentially enhances graft survival. VEGF can activate VEGFR-1, in turn promoting the expression of transcription factors involved in epithelial–mesenchymal transition (EMT). EMT can stimulate abnormal angiogenesis in HUVECs by activating the β-catenin-VEGF or Wnt/β-catenin pathway. In general, early-stage overexpression of VEGF increase islet transplantation success rate (69).

## 4 Cotransplantation of islets with a matrix

### 4.1 Cotransplantation with a hydrogel

Semi-permeable immunoprotective capsules containing therapeutic cells are a promising treatment option for metabolic disorders that require minute-by-minute metabolite regulation. Cell encapsulation has been used to effectively treat various diseases, including T1DM. To create the immunoprotective membranes, living cells are combined with biomaterials such as alginate (70), agarose (71), PEG, and polyvinyl alcohol (72) during the encapsulation process. This technique enables transplantation without using extreme immunosuppressive protocols, and it potentially addresses donor shortage through xenografting.

Encapsulation in immunoprotective membranes can enhance islet transplantation for diabetes treatment. The technique has demonstrated successful efficacy studies in rats, larger mammals, and even humans. However, the graft survival time remains consistently short—varying from days to months. Numerous factors, such as low oxygen supply, capsule biocompatibility, and inflammatory responses, may underlie the limited graft survival time. Therefore, using bioengineering technology to develop an encapsulation hydrogel can promote the activity of islets after islet transplantation and revascularization at the transplant site but without affecting the islet nutrient exchange and blood glucose sensing.

#### 4.1.1 Macroporous biohybrid cryogels

During the process of islet extraction, damage to the islets is inevitable, which can lead to a decrease in their activity. This process can also cause the separation of the islet ECM, making transplantation more difficult (73). Therefore, there is significant interest in improving transplanted islets’ survival and function. Researchers have focused on transplanting islets into nontraditional sites and preventing inflammation and cell death during and after transplantation. In particular, islet immunoisolation in animal models of diabetes is an active area of research, with a focus on encapsulating single insulin-producing β cells or entire islets using natural or synthetic biomaterials. Islet revascularization after transplantation is hampered by their encapsulation for immune response protection, which leads to graft hypoxia and attrition. Therefore, although the biocompatible material that encapsulates the islet facilitates immunoisolation, the material must neither affect the normal exchange of nutrients and oxygen between the islet and ECM nor block the islet-sensing blood glucose signaling pathway. Therefore, engineering additional optimal transplant microenvironments is essential for overcoming key challenges such as the lack of vasculature and ECM attachment or integrin ligation.

Macroporous cell scaffolds have attracted attention because of the large area of pathways for material exchange and insulin release, and the negative impact of the enclosed islets is small. Macroporous materials also tend to be more conducive to islet microvessel formation because of they provide relatively more convenient material exchange pathways. Borg et al. (74) developed a cryogel-based hydrogel system using star-shaped PEG and heparin cryogel. Their scaffolds feature a high porosity (approximately 98%) with thin yet robust struts withstanding deformation of around 90% without integrity loss. The local stiffness of the cryogel pore walls is much higher than the overall stiffness of the scaffold. This aids in protecting both MSCs and islets in the macropores while matching the tissue stiffness at possible extrahepatic transplantation sites.

The cryogel scaffolds have a Young’s modulus (0.6 kPa) similar to that of normal adipose and muscle tissue, making them suitable for subcutaneous implantation. Interconnected macropores enable unrestricted nutrition exchange while ensuring mechanical protection and 3D spatial distribution and retention of MSCs and islets. *In vitro*, the scaffolds allow for insulin production in response to glucose stimulation. Subcutaneous transplantation of these scaffolds in mice demonstrates the approach’s feasibility; however, due to technical limitations from fixation, islet function or insulin secretion have not been confirmed *in vivo*. The cryogel scaffolds offers protective support by utilizing a dual compensation mechanism to decrease ectopic apoptosis in pancreatic islets, with its feasibility demonstrated in short-term experiments. Nevertheless, limitations are present due to the limited duration of the study (74). Additional assessments of its long-term effects are necessary. Further experiments, research, and large-scale clinical trials are required for confirming its effectiveness and safety. This method necessitates more technological innovations and optimization to augment the reproducibility and consistency of outcomes and diminish the incidence of allograft rejection reactions. In summary, the cryogel scaffolds can be effectively used as scaffolding during the early stages of islet transplantation process.

#### 4.1.2 Semi-interpenetrating polymer network hydrogel

In practice, the implantation of cells immobilized in prefabricated hydrogels, such as macroporous biohybrid cryogels, typically requires surgery. Therefore, considerable emphasis has been placed on developing hydrogels that can form *in situ* after the injection of a liquid precursor solution. These injectable hydrogels offer several advantages, including minimizing patient discomfort as well as reducing infection and scar formation risks and treatment costs (75). Although injectable hydrogels can inject cells into specific subcutaneous sites, a lack of subcutaneous nutrition and oxygen can still reduce the function and survival of cells after transplantation.

Biomaterials containing methacrylic acid (MAA) possess inherent vascular regeneration properties without the need for exogenous factors such as cells or growth factors. Mahou et al. (26) developed a semi-interpenetrating polymer network (SIPN) by blending vinyl sulfone-terminated PEG (PEG-VS), and sodium polymethacrylate (PMAA-Na) with a stoichiometric amount of dithiothreitol; this SIPN can carry islet cells for injection into subcutaneous tissue and create a rich vascular network under the skin to promote transplanted islet cells’ survival. The authors examined three hydrogel formulations (1): PP8020 (80 mol% ethylene glycol + 20 mol% PMAA-Na) (2), PP9010 (90 mol% ethylene glycol + 10 mol% PMAA-Na), and (3) PEG (a 3D network formed using PEG-VS and DTT without any PMAA-Na). The swelling behavior of the SIPN hydrogels exhibited sensitivity to PMAA-Na, while only minor differences were noted in gelation time, permeability, and stiffness among the different hydrogel formulations. A vascular network with two or three times as many vessels was produced in the surrounding tissues by an SIPN containing 20 mol% PMAA-Na than by an SIPN containing 10 mol% PMAA-Na or PEG alone. *In vivo*, the glucose level steadily decreased and returned to normal levels by day 3 after 700 IEQ were injected into the dorsal flank of mice with streptozotocin-induced diabetes. Despite these findings illustrating the viability of cell delivery within a vascular, regenerating, injectable SIPN, additional studies on the effectiveness of PP8020 (an SIPN comprising 80 mol% ethylene glycol and 20 mol% PMAA-Na) over longer periods in various animals with diabetes. However, whether the vessels induced by PP8020 provide a benefit over PEG or other materials remains unclear.

The concept of using an injectable and inherently vascularizing semi-interpenetrating polymer network for delivering cells to the subcutaneous space is intriguing. This approach holds great promise for cell therapy delivery, particularly in the context of pancreatic islet transplantation. One notable advantage of this system is its injectability, enabling minimally invasive delivery to the subcutaneous space. The formation of a porous structure within the network facilitates the development of new blood vessels, thereby enhancing cell survival and function at the transplantation site. However, some considerations need to be addressed. It is crucial to evaluate the long-term persistence and degradation of the polymer network, ensuring that it does not elicit adverse effects or disrupt normal tissue function. Further studies are necessary to evaluate the immunogenicity and potential immune response associated with the use of polymer materials. Furthermore, determining the optimal composition and properties of the polymer network is essential to achieve consistent and reproducible outcomes. This includes optimizing mechanical strength, porosity, and biocompatibility. Thorough investigation is also needed to assess the compatibility and survival of different cell types within the network. In conclusion, while the concept of an injectable and vascularizing polymer network for cell delivery to the subcutaneous space shows promise, more research is required to overcome challenges and optimize this approach. It has the potential to revolutionize cell therapy treatments, but careful consideration of long-term safety, immunogenicity, and optimization of composition and properties is crucial for successful translation to clinical applications.

Hydrogel is a crucial component of islet transplantation that provides structural support and facilitates graft survival and vascular reconstruction during the early stages of subcutaneous transplantation. Controlled permeability is an essential characteristic when selecting hydrogels for cell delivery. With an appropriate pore size, the bidirectional diffusion of molecules such as oxygen and glucose, which are essential for cell survival, can be achieved and the permeation of immunoglobulin, leukocyte antigens, and antibodies can be prevented, thus providing immune protection and increasing cell survival. The stiffness of PEG hydrogels is adjustable. Moreover, biomaterials containing MAA enhance blood vessel reconstruction (76).

Hydrogels combining MAA and PEG may, therefore, facilitate subcutaneous device-free islet transplantation. These hydrogels—which not only regulate stiffness but also facilitate blood oxygen reconstruction in subcutaneous islet transplantation—are more mature than hydrogels that mainly play the role of scaffolds.

#### 4.1.3 Degradable MAA-based synthetic hydrogel

Synthetic MAA-based biomaterials produce numerous blood vessels in the subcutaneous area without the need for other biologicals (76). Kinney et al. (25) encapsulated islets in a naturally vascularized MAA hydrogel and then transplanted them into the subcutaneous membrane of streptozotocin-induced diabetic mice; they were noted to result in mature, well-vascularized subcutaneous tissue. Moreover, the islets could override hyperglycemia and endure for as long as 70 days. The authors also created a degradable version of the MAA-PEG polymer network and proposed that when an islet-containing, naturally vascularized, biodegradable MAA-PEG hydrogel was injected into the subcutaneous site, the islets demonstrated stabilized survival in the subcutaneous area. This procedure was performed without using vascularized cells or growth factors. Mice with streptozotocin-induced diabetes demonstrated stable blood sugar levels for 70 days after receiving an injection of 600 IEQ encased in an MAA-PEG hydrogel. A well-vascularized matrix replaced the MAA-PEG hydrogel scaffold to support subcutaneously transplanted islets. The matrix deteriorated over a short period. However, the findings indicated that within 3–7 days before hydrogel degradation, the MAA material primed a series of vascularized endogenous healing mechanisms, enabling subcutaneous graft implantation. During the disappearance of the hydrogels, the body produced enough subcutaneous tissue to support the graft (25).

During islet delivery, hydrogel degradation control is crucial. Rapid degradation may lead to the release of unanchored islets, leading to a hostile inflammatory host response that makes engraftment difficult, whereas delayed degradation may impede islet integration and cause islet death through anoikis-like apoptosis, before revascularization occurs; this is because of the disconnection of islet–ECM interactions and inadequacy of nutrient diffusion. Synthetic hydrogels provide an advantage: islet engraftment timelines can be regulated by tuning material properties, including degradation. For instance, administering islets in nondegradable or slow-degrading MAA-PEG hydrogels reduces the average daily blood glucose levels compared with fast-degrading MAA-PEG hydrogels, indicating the importance of controlling degradation for successful islet engraftment (25).

MAA-PEG encapsulation materials are synthetic biomaterials that can induce the formation of numerous blood vessels in the subcutaneous space, even without the use of additional biologics. This phenomenon is attributable, at least partly, to the ability of MAA-PEGs to stimulate the polarization of host macrophages toward a proregenerative state through the activation of the sonic hedgehog pathway, the insulin growth factor pathway, or both (77). PEG hydrogels, which are biocompatible, protein-repellant, and nonimmunogenic, exhibit highly customizable physical properties, including tunable stiffness through precursor concentration or arm length selection. Hydrogel degradation rates can be controlled by manipulating PEG crosslink density (78). MAA-PEG hydrogels have some properties that make them inherently suitable and injectable for a straightforward, noninvasive one-step injection process for subcutaneous islet transplantation. This results in a reduction in inflammation related to transplantation and a reduction in the amount of resources required per patient and logistical constraints associated with the procedure. Consequently, this approach enhances the clinical feasibility of islet transplantation. These characteristics indicate that MAA-PEG biomaterials are relatively mature hydrogels, which can be applied to subcutaneous islet transplantation. However, additional experiments determining the effects of subcutaneous transplantation of MAA-PEG–coated islets on the production of subcutaneous vascularized tissue are warranted.

The development of degradable methacrylic acid-based synthetic hydrogels for subcutaneous islet transplantation is a promising approach to address the challenges of traditional islet transplantation, such as the need for immunosuppressants and the limited supply of donor islets.One advantage of using synthetic hydrogels is their tunable mechanical and degradation properties, which can be controlled to match the physiological requirements of islet transplantation (79). Additionally, synthetic hydrogels are biocompatible and can be functionalized with specific biomolecules to improve their performance, such as cell adhesion or angiogenesis.However, there are still some challenges to overcome. The degradation profile of the hydrogel should be optimized to ensure that the islets survive and thrive in the transplantation site, while the hydrogel gradually degrades without accumulating deleterious byproducts in the host. The long-term safety and immune compatibility of the hydrogel must also be evaluated. In conclusion, the development of degradable synthetic hydrogels for subcutaneous islet transplantation is a promising approach. However, further research is necessary to optimize the hydrogel’s properties, evaluate its long-term safety and immune compatibility, and refine the method of hydrogel delivery. With continued research and refinement, this approach may offer a viable alternative to traditional islet transplantation and revolutionize diabetes treatment.

#### 4.1.4 Arg-Gly-Asp–modified injectable hydrogel

ECM breakdown during islet extraction and purification steps leads to islet damage and dysfunction; therefore, researchers have focused on developing 3D ECM structures mimicking the internal environment of islets to improve islet function. These 3D structures facilitate the development of cell-to-cell and cell-to-ECM connections, required for cell differentiation, cell regeneration, and apoptosis prevention (80).

Collagen and laminins are crucial connective molecules for islet function in the ECM. The peptide Arg-Gly-Asp (RGD), also present in various ECMs, is the most extensively investigated laminin. RGD in alginate semipermeable capsules at the right dose can enhance islet function *in vitro* (81, 82). In addition, because of its body temperature–induced coacervation capabilities, RGD is ideal for use *in vivo* and *in vitro* (83).

Lan et al. (84) examined the effects of VitroGel, a 3D hydrogel, on β-TC6-cells, frequently used in the development of new medications and the study of the disease mechanisms. The authors clarified the impact of RGD alteration on β-cell survival and function and explored the impacts of various concentrations of the 3D hydrogel. Moreover, *in vitro* cell proliferation and survival rates and the *in vivo* tissue volume were used to assess cell viability. Furthermore, their roles in insulin secretion and blood glucose regulation were assessed. Finally, the authors reported that the RGD–3D hydrogel maintained cell function and promoted their proliferation.

The utilization of RGD-modified injectable hydrogel for maintaining islet beta-cell survival and function in subcutaneous transplantation is a promising strategy in the field of islet transplantation.One advantage of using RGD-modified hydrogel is its ability to provide a supportive microenvironment for the transplanted islet beta cells (85). The inclusion of the RGD (arginine-glycine-aspartic acid) peptide sequence in the hydrogel promotes cell adhesion and interaction with the extracellular matrix, allowing for improved cell survival and function.In addition to enhancing cell adhesion, the injectable hydrogel provides a scaffold that assists in the organization and integration of the transplanted islet beta cells. This can potentially enhance their overall functionality and enable better glucose regulation.However, there are some considerations to address. Further research is needed to fully understand the long-term effects of the RGD-modified hydrogel on the transplanted cells and the host tissue. With continued development, this approach may contribute to the advancement of effective and efficient islet transplantation therapies for diabetes treatment.

### 4.2 Cotransplantation with simulated ECM

#### 4.2.1 Agarose-SEK-1005 rods

A considerable amount of evidence related to subcutaneous islet transplantation has been reported recently. When subcutaneous transplant locations are used, transplanting and removing islets while under local anesthesia is easier. *Streptomyces nobilis* produces the cyclic oligopeptide SEK (86), which might represent a new class of anti-inflammatory medication. SEK may accelerate full-thickness wound healing (87), and it is a strong inducer of TGF-β1. When allogeneic islets were placed in prevascular subcutaneous locations and treated with agarose-fibroblast growth factor-2 in a rat model of T1DM, prolonged survival and euglycemia maintenance without immunosuppressive treatment were noted (86). The authors indicated that SEK might favor the production of a rich vascularized space in subcutaneous tissue during islet transplantation. They also examined the survival rate of allogeneic islet transplantation without immunosuppressive therapy. Notably, SEK was noted to induce VEGF and TGF-β1 production in the HUVEC environment, in turn encouraging the development of microvessels in collagen gels. Notably, SEK did not directly cause VEGF synthesis; instead, it induced the release of TGF-β1, followed by that of VEGF, in HUVECs. TGF-β1 could also induce Tregs, resulting in the formation of immune-tolerant, highly vascularized granulomatous tissue. Based on the nonfasting blood glucose levels, this technique supported long-term graft survival without immunosuppressive use in 8 out of 10 mouse recipients.

Islet transplantation studies, both clinical and experimental, have explored several possible transplantation sites. These sites are required to meet certain criteria: abundant blood supply, easy access through minimally invasive procedures, and a clear view of islet graft function and appearance. In a recent study (86), we found that implanting agarose-SEK rods in the subcutaneous tissue created an immune-tolerant environment surrounded by connective tissue with blood vessels; this site fulfilled most of the aforementioned criteria. However, because agarose-SEK rods need to be implanted into the skin of the recipient’s back before islet transplantation, the entire islet transplantation process requires two surgical procedures; this increases the inflammatory response at the transplant site as well as the instability of clinical application. Thus, additional studies on whether the generated site and methodology are appropriate for clinical application are required.

#### 4.2.2 Fibrin scaffold

Using a near-native ECM can help to avoid hypoxia and boost local vascularization (27). In an ectopic site, the ECM is essential for graft survival because islet survival is greatly influenced by contact between cell surface receptors and ECM components. *In vitro*, the matrix fibers’ 3D configuration enhances cell attachment and inhibits islet cell dedifferentiation. However, the process of islet extraction can negatively affect the islet ECM; therefore, before transplantation of the extracted islets, some bioengineering techniques should be undertaken to rebuild the islet ECM and maintain islet function and activity. Hydrogels and some other biocompatible bioscaffolds are effective technical means to rebuild the islet microenvironment and protect the islets.

Fibrin, a crucial component of hemostasis and wound healing (88), is a proangiogenic plasma transglutaminase with a proliferative effect on fibroblasts (89). Moreover, RGD, found in fibrin and other ECM components, interacts with cell surface integrins to accelerate cell maturation and function. Fibrin also has been demonstrated to enhance β-cell engraftment in the subcutaneous area. Moreover, it is biocompatible and biodegradable, and it can crosslink *in vitro* and *in vivo*, allowing cell seeding and promoting graft distribution.

Salama et al. (27) reported that subcutaneous transplantation of fibrin increases angiogenesis, improves NPI graft survival and long-term function, and ultimately, normalizes blood glucose levels in mice. Engraftment of NPIs combined with fibrin in the subcutaneous area, without requiring proangiogenic growth factors or prevascularization, is effective due to the resistance of NPIs to hypoxia, human proinflammatory cytokines, and hyperglycemia. De Mesmaeker et al. recently demonstrated that alginate-encapsulated porcine prenatal β cells, when transplanted subcutaneously, generate a functional β-cell mass effectively but human islets do not (27). The authors attributed this to the immature state of the porcine prenatal β cells and suggested that immature porcine β cells are less metabolically active and have lower oxygen requirements, making them better fitted to a hypoxic microenvironment. In contrast, mature β cells are more metabolically active and vulnerable to hypoxia; their survival requires vascularization.

Therefore, the role of fibrin as the ECM of islet transplantation and the validity of the two-step surgical method (preimplantation of fibrin stent in the subcutaneous area followed by islet transplantation) in clinical practice warrant further investigation

#### 4.2.3 Device-free islet viability matrix

For islets to survive, their engraftment environment is crucial. Studies have clarified the significance of collagen I in the peri-islet extracellular milieu of a healthy mammalian pancreas (90). The milieu favorable for graft following subcutaneous islet transplantation can be effectively restored by using oligomeric or encapsulated collagen matrices (42, 91). Human collagen I, L-glutamine, fetal bovine serum, sodium bicarbonate, and medium 199, in specific proportions, have been configured into an islet active matrix; when this matrix is combined with mouse, pig, or human islets for subcutaneous transplantation, the uniform survival of pancreatic islets increases (92).

Simple, safe, and repeatable islet–islet viability matrix (IVM) subcutaneous transplantation represents a new T1DM therapy paradigm. At present, portal islet delivery is the most commonly used clinical application; however, the research results of subcutaneous islet transplantation combined with IVM have resulted in this protocol gradually becoming an important choice for islet transplantation. Studies have shown that the glucagon-like peptide 1 receptor signaling pathway can upregulate B-cell CLL/lymphoma 2 (BCL2); however, BCL2 has an antiapoptotic effect, which might be a mechanism through which IVM enhances cell function after subcutaneous islet transplantation (**Table 2**) (92). IVM-admixed islets maintain consistent insulin and glucagon production by sustaining islet viability in the subcutaneous compartment. It can strengthen the case for the cotransplantation of IVM combined with stem cell–derived cells and improve the clinical use of islet transplantation, particularly allogeneic islet transplantation, considerably.

**Table 2:** Introduction to biomaterials.

Biomaterials	Preparation method	Advantage	Supplement	Reference
Macroporous biohybrid cryogel	Macroporous, star-shaped poly(ethylene glycol) (starPEG)-heparin cryogel scaffolds are covalently modified with adhesion peptides.	Provides mechanical protection Enables exchange of oxygen, nutrients, and metabolites Produces natural islet microenvironment	Macroporous biohybrid cryogels play a supporting role. MSCs adhering to hydrogels build a natural islet microenvironment.	(74)
Semi-interpenetrating polymer network (SPIN) hydrogel	A blend of vinyl sulfoneterminated polyethylene glycol (PEG-VS) is reacted with sodium polymethacrylate (PMAA-Na).	Has controlled permeability and hardness Enhances vascularization	SIPN containing 20 mol% PMAA-Na generates a vascular network in the surrounding tissues, with two to three times as many vessels as those obtained with 10 mol% PMAA-Na or PEG alone.	(26)
Agarose-SEK-1005 (SEK)	A cyclic oligopeptide, SEK, is isolated from *Streptomyces nobilis*	Accelerates healing of full-thickness wounds Acts as a potent inducer of TGF-β1 and VEGF Facilitates highly vascularized, immune-tolerant environment formation	Its preparation involves requires two steps: (i) creation of a richly vascularized space for islet transplantation in the subcutaneous tissue and (ii) transplantation of islets.	(62)
Arg-Gly-Asp (RGD)-modified injectable hydrogel	It is produced via RGD modification on VitroGel 3D hydrogel.	Maintains β-cell proliferation and vascularization, can be used in vivo, and has adjustable mechanical properties	/	(84)
Fibrin scaffold	Fibrin-alone scaffolds are constructed using a commercially available fibrin sealant kit.	Facilitates hemostasis and wound healing Is biocompatible and biodegradable Facilitates graft delivery	/	(27)
Device-free islet viability matrix (IVM)	It is created using a blend of 10x M199, L-glutamine, FBS 7.5% sodium bicarbonate, NaHCO3, and type I collagen.	Induces islet adhesion Inhibits inflammation and apoptosis, Facilitates neovascularization Elicits prosurvival signals Downregulates apoptosome activity	It has demonstrated efficiency in cynomolgus monkeys	(50)
Degradable methacrylic acid–based synthetic hydrogel	It is created by combining a semi-interpenetrating polymer network of PMAA-Na and PEG.	Generates numerous blood vessels Improves nerve regeneration and tissue repair Is degradable	MAA-PEG degradation rate affects graft survival.	(25)

#### 4.2.4 Poly (lactic-co-glycolic acid)

In addition to the above methods, microspheres made of polymer also have the function of islet delivery. The use of particulate-based biomaterials has allowed researchers to achieve the best immunosuppressive effects. Recent research (93) has shown that co-delivery of FK506-loaded polymeric microspheres with islets significantly improved graft survival. A biodegradable and biocompatible poly(lactic-co-glycolic acid) was used as the drug delivery vehicle to ensure sustained drug release. However, premature rejection of islet grafts in some recipients motivated the development of an effective immunosuppressive protocol using a single-dose of FK506-loaded microspheres and clodronate liposomes. One significant advantage of this approach is its ability to directly target and modulate the immune cells in the immediate area surrounding the transplant site. By delivering immunosuppressive agents locally and in a controlled manner, it may be possible to induce a tolerogenic immune environment that facilitates acceptance of the transplanted cells. Additionally, the use of tolerogenic dendritic cells is a valuable asset, as they play vital roles in promoting immune tolerance and generating regulatory T cells (94). This action can help to further suppress alloreactive immune responses and enhance graft tolerance. Additionally, the use of tolerogenic dendritic cells is a valuable asset, as they play vital roles in promoting immune tolerance and generating regulatory T cells. Further research and development are necessary to optimize the approach and address any potential safety concerns before clinical translation becomes feasible.

The polymeric microsphere-based site-specific delivery of quercetin also is a promising strategy for preventing senescence of pancreatic islets and improving transplantation outcomes in diabetes mouse models (95). This approach offers advantages such as targeted delivery of quercetin to the islets, which can improve therapeutic efficacy while minimizing systemic side effects. Quercetin, a natural antioxidant and anti-inflammatory agent, can potentially protect islets from oxidative stress and age-related damage, thus enhancing islet survival and function after transplantation. Meanwhile, ensuring the selection of appropriately sized islets is essential to improve the transplantation outcome. To mitigate the issue of islet death due to hypoxia after transplantation, they propose using size-controlled ICCs (Islet Cell Clusters) as a potential solution (96). Through their study, they prioritized optimizing the size of ICCs using the hanging-drop technique within a hypoxic culture environment. Their subsequent investigation involved transplanting these clusters into the subcutaneous space of BALB/c nude mice to assess their functionality and viability. The viability of ICCs and control islets under normoxia was similar, while ICC500 and ICC1000 exhibited minimal cell death in a hypoxic environment. Transplanting ICCs within Matrigel resulted in improved transplantation outcomes, underscoring the importance of separating larger islets before generating size-controlled ICCs to prevent loss during transplantation. Microencapsulation of ICCs is shown to be effective in combating immune rejection and hypoxia; however, islet necrosis remains a concern due to the expansion of the islet diameter after encapsulation. Moreover, the number and mass of transplanted islets correlated with maintaining euglycemia, with ICCs demonstrating superior *in vivo* function compared to control islets in immune-compromised mice. Gene transduction can further enhance ICC functions and improve therapeutic outcomes. Overall, this study (96) highlights ICC transplantation’s potential as a promising strategy for islet transplantation therapy, with further strides made towards its optimization through upcoming research. It is evident that the success of pancreatic islet transplantation relies not only on the reconstruction of the islet microenvironment but also on the size of the graft. Furthermore, this approach has the potential to address one of the major challenges in islet transplantation – lack of healthy islet cells for transplantation. The technique’s ability to prevent islet senescence can help to make better use of available donor islets for transplantation while expanding the donor pool. Despite its many potential benefits, further research is needed to optimize this approach and study its safety. Researchers need to determine the ideal degree and duration of quercetin delivery, as well as investigate the mechanism of action, to ensure optimal therapeutic efficacy while maintaining safety. In conclusion, the polymeric microsphere-based site-specific delivery of quercetin shows promise in preventing islet senescence and improving transplantation outcomes in diabetes mouse models. Researchers need to continue researching and developing this approach to maximize its potential therapeutic benefits and optimize its delivery and safety.

## 5 ECM regulation after subcutaneous device-free islet transplantation

Subcutaneous device-free islet transplantation requires modulation of ECM components to protect and improve islet function and survival because of a lack of adequate revascularization, autoimmune recurrence, and immediate blood-mediated inflammatory response. Here, we summarize three strategies for regulating ECM.

### 5.1 Decellularized ECM scaffolds

The decellularized organ ECM (97) is frequently used for tissue engineering and islet transplantation (94). A major advantage of using decellularized ECM structures is the significant decrease in immunogenicity achieved through cellular material removal. As such, ECM scaffolds form an ideal support system for transplanted cells. The pancreas is theoretically an ideal candidate for decellularization in islet transplantation because it closely resembles the native pancreatic ECM. However, *in vivo* data supporting this concept are lacking.

Abualhassan et al. infused 500 mouse islets into decellularized lung tissue and transplanted them into the peritoneal cavity of mice with diabetes (98). The author noted normoglycemia in 67% of the mice when an ECM scaffold was used; however, it was noted in only 13% of the mice when the ECM scaffold was not used.

In a study (98) involving human islets, promising results were observed: Diabetes remission was achieved through the transplantation of 1000 human IEQ into decellularized lung tissue. However, the study could not sustain normal blood glucose levels until the end of the study. This method is tedious and difficult, and its clinical application value may be relatively limited.

### 5.2 Artificial ECM

Incorporating particular ECM molecules can viably extend the functional lifespan of islets. This approach combined with immunoisolation strategies, such as islet encapsulation, has demonstrated efficacy (99–101). Enclosing islets within a semipermeable membrane, which shields it against immune reactions, enabling effective transplantation without the need for immunosuppressive treatment. However, selecting the appropriate ECM molecules and concentrations is crucial because not all molecules are beneficial to islet function, and ECM molecules at high concentrations may even be detrimental to the islet cells. For instance, collagen IV at extremely high concentrations negatively impacts glucose-induced insulin secretion (101). Furthermore, only specific laminin sequences (RGD, LRE, and PDSGR) in combination with collagen IV have a positive effect on human islet function. In particular, the laminin sequences RGD, LRE, and PDSGR can enhance glucose-induced insulin release *in vitro* (102). Cotransplantation of islets with a simulated ECM is an approach to improve posttransplantation islet function and graft viability.

### 5.3 *In vivo* stimulation of ECM production

Other techniques that supplement ECM components, such as restoring the islet ECM entirely, are available. Fibroblasts that produce the native ECM in and around the islets in the pancreas can be cocultured to improve islet function and create and preserve a fibronectin-rich ECM (103). Islet grafts may also benefit from being cocultured with MSCs that secrete fibronectin and laminin (51).

Another technique involves *in vivo* restoration of the ECM via the injection growth factors such as HGF, VEGF, and TGF-β, which can stimulate fibroblast proliferation and encourage fibronectin and collagen production, thus facilitating tissue repair around the transplanted islets (103). Connective tissue growth factor may also stimulate ECM production (73).

## 6 Conclusions

Islet transplantation research includes the study of the environment, ECM content, tissue-resident immune cells, and graft angiogenesis at the transplant site. Islet grafts cannot survive in the intrahepatic environment over the long term; this limits the site’s potential for use in islet transplantation in patients with T1DM. In contrast, subcutaneous device-free islet transplantation is an attractive approach for treating type 1 diabetes, with several advantages over traditional pancreas transplantation. Researchers are actively exploring ways to improve the success of this procedure. This article discussed our perspective on subcutaneous islet transplantation, focusing on the use of other cells, growth factors such as HGF and VEGF, and various hydrogels and scaffolds for cell encapsulation, including Agarose-SEK-1005 (SEK) rods, fibrin scaffold, device-free islet viability matrix and PLGA. To begin, adMSC, bMSC, and endothelial cells have shown great potential in enhancing the success of subcutaneous islet transplantation. Co-transplantation of these cells with islets improves vascularization, reduces inflammation, and enhances islet survival and function. Additionally, these cells provide long-term support for the islets due to their immune-privileged nature. Incorporating growth factors like HGF and VEGF plays a crucial role in promoting angiogenesis and improving islet survival, function, and insulin secretion. Co-transplantation of these growth factors with islets has shown promising results in enhancing the success of subcutaneous islet transplantation. The use of different types of hydrogels and scaffolds for cell encapsulation offers a favorable environment for islets. Macroporous biohybrid cryogels, semi-interpenetrating polymer network hydrogels, degradable MAA-based synthetic hydrogels, and Arg-Gly-Asp–modified injectable hydrogels have demonstrated success in improving the outcomes of subcutaneous islet transplantation. Various scaffolds have been developed to provide support for islet transplantation. Agarose-SEK-1005 (SEK) rods, fibrin scaffold, device-free islet viability matrix, and PLGA have been explored as potential options. For example, SEK rods have shown high islet viability and function, while PLGA holds promise in providing long-term support for the islets.

In conclusion, subcutaneous islet transplantation holds potential in revolutionizing the treatment of type 1 diabetes. However, thorough research and clinical trials are necessary to ensure safety and efficacy. The use of advanced techniques in subcutaneous islet transplantation can significantly improve the quality of life for millions of diabetes patients worldwide, and it is important to continue exploring new approaches and strategies for this promising treatment option. As medical professionals, we should stay up-to-date with the latest advancements in the field and work towards finding ways to improve the success of subcutaneous islet transplantation.

Furthermore, cell encapsulation technology plays a crucial role in islet transplantation. ViaCyte, Inc. is the most advanced in cell therapy encapsulation, with a multilayer design that uses a semipermeable polytetrafluoroethylene membrane and an external polyester mesh to protect implanted cells (104). PEC-01 is a therapy that involves directing human embryonic stem cells to differentiate into pancreatic endoderm cells (105). This therapeutic approach utilizes the encapsulation technology of the Encaptra^®^ immune protection device, which is implanted under the patient’s skin. Once successfully implanted, these cells mature into insulin-secreting β cells, effectively regulating blood sugar levels. Furthermore, they also have the capability to generate other normal pancreatic cells. However, their VC-01 drug candidate, which used the “PEC-Encap” device to protect cells from immune responses, experienced biomaterial-elicited fibrosis in humans that limited cell survival and did not produce C-peptide in patients in 2014(NCT02239354). To address this issue, ViaCyte, Inc. is collaborating with Gore-Tex to develop a modified PEC-Encap device with increased biocompatibility to avoid fibrotic responses (106). Meanwhile ViaCyte Inc. is collaborating with CRISPR Therapeutics to optimize the use of the PEC-Direct device by using genetically edited cells to evade immunity. They plan to delete the β2-microglobulin gene and transgenically express PDL-1, among other undisclosed gene edits, to enable the use of the PEC-Direct device without requiring chronic immune suppression (107). The clinical trial (NCT05210530) is currently ongoing, and ViaCyte, Inc recently reported in a February 2022 press release that the first patient in the trial received implanted cells. Successful generation of immune-evasive universal donor cells may eventually eliminate the need for any form of encapsulation during implantation. One of the advantages of ViaCyte’s technology is the ability to provide a potential cure for type 1 diabetes by replacing the lost or damaged islet cells that produce insulin. Additionally, the subcutaneous implantation of the encapsulated cells avoids the need for systemic immunosuppression therapy and reduces the risk of immune system rejection. Furthermore, the use of stem cell-derived progenitor cells as a source for the encapsulated cells offers several advantages, including a potentially unlimited supply of cells, easier scalability, reduced risk of contaminating pathogens, and lower cost. However, there are still challenges to overcome regarding the scalability and production of the encapsulated cells, as well as addressing potential complications such as fibrosis and vascularization of the implant site.

Device-free subcutaneous islet transplantation eliminates the need for an encapsulation device. This streamlined approach simplifies the procedure, reduces costs, and potentially minimizes the risk of device-related complications, such as infection or mechanical failure. Additionally, device-free transplantation may facilitate a more natural interaction between the transplanted islets and surrounding tissues, potentially promoting improved integration and functionality.On the other hand, device-based encapsulation has emerged as a promising strategy for improving subcutaneous islet transplantation. Encapsulation involves the use of a protective barrier that shields the transplanted islets from the host’s immune system, ultimately preventing rejection and the need for immunosuppressive drugs. This approach offers several advantages over device-free transplantation.Firstly, encapsulation provides a physical barrier that isolates the transplanted islets from surrounding tissues. This effectively reduces the risk of immune cell infiltration and subsequent destruction of the islets, resulting in improved survival and functionality. Furthermore, the encapsulation device enables controlled and sustained release of therapeutic factors like growth factors and immunomodulatory agents, which can further enhance the survival and function of the transplanted islets.Moreover, encapsulation devices offer the benefit of retrievability. In the event of any adverse effects or complications, the device can easily be removed, allowing for necessary adjustments or modifications as required. This flexibility ensures personalized treatment tailored to each patient’s specific needs and evolving conditions over time. With the advancement of technology, the choice between device-based encapsulation and device-free subcutaneous islet transplantation will largely depend on individual patient factors. Factors such as the extent of autoimmunity, the potential for immune rejection, and the requirements for long-term immunosuppression will all play a role in determining the optimal approach. A personalized treatment strategy, which takes into account aspects such as disease progression, patient immune status, and individualized risks and benefits, will be crucial for maximizing treatment outcomes. In conclusion, device-based encapsulation offers significant advantages in terms of immune protection and controlled release of therapeutic factors, albeit with potential device-related complications. On the other hand, device-free subcutaneous islet transplantation streamlines the procedure and offers integration benefits. As we progress, a comprehensive evaluation of each patient’s unique circumstances will be pivotal in deciding the most suitable approach for subcutaneous islet transplantation.

## Author contributions

XZ: Writing – original draft. ZX: Writing – review & editing. YaY: Writing – review & editing. WY: Writing – review & editing. BF: Writing – review & editing. YuY: Writing – review & editing. FL: Writing – review & editing. JC: Writing – review & editing. HG: Writing – review & editing.
